# Beyond Static Benchmarking: Using Experimental Manipulations to Evaluate Land Model Assumptions

**DOI:** 10.1029/2018GB006141

**Published:** 2019-10-28

**Authors:** William R. Wieder, David M. Lawrence, Rosie A. Fisher, Gordon B. Bonan, Susan J. Cheng, Christine L. Goodale, A. Stuart Grandy, Charles D. Koven, Danica L. Lombardozzi, Keith W. Oleson, R. Quinn Thomas

**Affiliations:** ^1^ Climate and Global Dynamics Laboratory National Center for Atmospheric Research Boulder CO USA; ^2^ Institute of Arctic and Alpine Research University of Colorado Boulder Boulder CO USA; ^3^ Department of Ecology and Evolutionary Biology Cornell University Ithaca NY USA; ^4^ Department of Natural Resources and the Environment University of New Hampshire Durham NH USA; ^5^ Climate and Ecosystem Sciences Division Lawrence Berkeley National Laboratory Berkeley CA USA; ^6^ Department of Forest Resources and Environmental Conservation Virginia Tech Blacksburg VA USA

**Keywords:** Community Land Model, nitrogen enrichment, elevated CO_2_, land model, biogeochemistry

## Abstract

Land models are often used to simulate terrestrial responses to future environmental changes, but these models are not commonly evaluated with data from experimental manipulations. Results from experimental manipulations can identify and evaluate model assumptions that are consistent with appropriate ecosystem responses to future environmental change. We conducted simulations using three coupled carbon‐nitrogen versions of the Community Land Model (CLM, versions 4, 4.5, and—the newly developed—5), and compared the simulated response to nitrogen (N) and atmospheric carbon dioxide (CO_2_) enrichment with meta‐analyses of observations from similar experimental manipulations. In control simulations, successive versions of CLM showed a poleward increase in gross primary productivity and an overall bias reduction, compared to FLUXNET‐MTE observations. Simulations with N and CO_2_ enrichment demonstrate that CLM transitioned from a model that exhibited strong nitrogen limitation of the terrestrial carbon cycle (CLM4) to a model that showed greater responsiveness to elevated concentrations of CO_2_ in the atmosphere (CLM5). Overall, CLM5 simulations showed better agreement with observed ecosystem responses to experimental N and CO_2_ enrichment than previous versions of the model. These simulations also exposed shortcomings in structural assumptions and parameterizations. Specifically, no version of CLM captures changes in plant physiology, allocation, and nutrient uptake that are likely important aspects of terrestrial ecosystems' responses to environmental change. These highlight priority areas that should be addressed in future model developments. Moving forward, incorporating results from experimental manipulations into model benchmarking tools that are used to evaluate model performance will help increase confidence in terrestrial carbon cycle projections.

## Key Points

- Experimental manipulations provide critical insights into ecosystem responses to environmental change that can evaluate land models
- Parametric and structural changes to the Community Land Model version 5 improve the simulated response to environmental change
- Model assumptions related to nutrient acquisition strategies and trade‐offs between carbon and nitrogen limitation deserve further attention

## 1 Introduction

Large uncertainties in terrestrial carbon (C) cycle projections presents significant barriers to developing greenhouse gas emissions that are compatible with particular climate change scenarios (Friedlingstein et al., 2014; Jones et al., 2013). In particular, the projected concentrations of atmospheric CO_2_ and the magnitude of carbon‐climate feedback shows persistently high uncertainty that is largely related to structural uncertainty among land models (Arora et al., 2013; Friedlingstein et al., 2006; Lovenduski & Bonan, 2017). Indeed, since the IPCC Fifth Assessment Report, significant efforts have gone into understanding model deficiencies, improving the representation of particular land processes, and developing the current generation of land models that will be used in upcoming climate change assessments (Eyring et al., 2016; Jones et al., 2016; Lawrence et al., 2016). Some of these advances include addition or modifications of land model simulations of the nitrogen (N) cycle and its constraints on terrestrial carbon balance (Thornton et al., 2007; Zaehle et al., 2010). As the science behind Earth system models matures, it creates a need to develop uniform metrics that can be used to evaluate model performance and to understand what model assumptions lead to appropriate, or likely, responses to environmental change.

Model benchmarking tools, like the International Land Model Benchmarking Project (ILAMB), offer powerful insights into the biogeophysical and biogeochemical representations of land models (Collier et al., 2018; Hoffman et al., 2017; Luo et al., 2012). By standardizing the observational data sets and scoring metrics by which models are evaluated, researchers gain understanding of model strengths and deficiencies (Lawrence et al., 2019). While matching historical trends and present‐day observations are an important prerequisite for credible land model simulations, they offer relatively little insight into the accuracy of future projections that such models are intended to make. Given that future environmental changes will likely exceed the range of historical conditions, improvements in benchmarking scores over the historical era do not necessarily improve confidence in future projections.

To gain a greater understanding of ecological processes that are represented in models we need to move beyond these static benchmarks. Evaluating terrestrial models against observed responses to experimental manipulations (e.g., atmospheric CO_2_ enrichment) helps to identify and evaluate assumptions that are important for simulating appropriate ecosystem responses to future environmental change (De Kauwe et al., 2014; Medlyn et al., 2015; Walker, Hanson, et al., 2014; Zaehle et al., 2014). When experimental manipulations are replicated in models, the outcomes provide additional insights into model strengths and weaknesses that will influence future projections and could be incorporated into benchmarking packages like ILAMB. Toward this end, we conducted simulations using three coupled carbon‐nitrogen (C‐N) versions of the Community Land Model (CLM), the land component of the Community Earth System Model, and compared the global response to nitrogen and CO_2_ enrichment simulated by the models to meta‐analyses from similar experimental manipulations.

The initial representations of terrestrial biogeochemical cycles in early climate models simulate leaf gas exchange and plant productivity based on Ball‐Berry stomatal conductance and Farquhar photosynthesis, respectively (Bonan, 2015). Although implementation and canopy scaling of these schemes among models remains uncertain (Rogers et al., 2017), they typically project a strong carbon‐concentration feedback and robust terrestrial carbon sink under elevated CO_2_ (Arora et al., 2013). These carbon‐only models potentially exaggerate the magnitude of the land carbon sink (Hungate et al., 2003; Wieder, Cleveland, Smith, & Todd‐Brown, 2015; Zaehle et al., 2015), thus motivating the representation of the terrestrial nitrogen cycle into land models, which typically reduces ecosystem sensitivity to elevated CO_2_ and decreases terrestrial carbon uptake in future scenarios (Thornton et al., 2007; Wang & Houlton, 2009; Zaehle et al., 2010). Alternatively, inclusion of coupled C‐N dynamics can also offset or even reverse the direction of the carbon‐climate feedback, when warming‐enhanced mineralization of N from soil organic matter stimulates more CO_2_ uptake by plants than is lost to warming‐enhanced decomposition (Sokolov et al., 2008). Although the inclusion of coupled C‐N dynamics into global‐scale models affords opportunities to integrate ecological and biogeochemical insights into historically geophysical models, it also creates significant uncertainties in model structure and parameterization that underscore our incomplete understanding of terrestrial ecosystem function and how to represent them at global scales. For example, the mechanisms by which nitrogen fundamentally limits terrestrial productivity remain uncertain (Meyerholt & Zaehle, 2015; Meyerholt & Zaehle, 2018; Thomas et al., 2015). Similarly, the magnitude and duration of ecosystem response to elevated CO_2_ remains poorly constrained (De Kauwe et al., 2014; Zaehle et al., 2014). This paper aims to quantify sensitivities to nitrogen enrichment and elevated CO_2_ in three coupled C‐N versions of CLM. Despite their structural similarities, over the course of model development each version of CLM makes different assumptions about plant and soil biogeochemical processes that lead to different responses to N enrichment and elevated CO_2_. Here we evaluate these assumptions with data from experimental manipulations that increase N and CO_2_ availability and suggest that similar activities afford opportunities to extend insights from future model intercomparison projects.

## 2 Methods

### 2.1 Model Overview

In the Coupled Model Intercomparison Project phase 5 (CMIP5) ensemble of models, the Community Land Model, version 4 (CLM4) was the only terrestrial model that included coupled carbon‐nitrogen biogeochemistry (Thornton et al., 2007). This version of the model showed strong nitrogen limitation (Bonan & Levis, 2010; Thomas, Zaehle, et al., 2013) and a weak response to elevated CO_2_ (Hoffman et al., 2014; Zaehle et al., 2014). The intermediate model version, CLM4.5 (Oleson et al., 2013), implemented modifications to canopy photosynthesis (Bonan et al., 2011; Bonan et al., 2012) and a vertically resolved representation of soil biogeochemistry, which resulted in a twentieth‐century land carbon sink that better matched observationally derived estimates (Koven et al., 2013), but still showed strong nitrogen limitation of the global carbon cycle (Wieder, Cleveland, Lawrence, & Bonan, 2015). Finally, CLM5 implemented extensive modifications to the representation of plant nitrogen dynamics (Fisher et al., 2019), snow and soil hydrology (Brunke et al., 2016; Swenson & Lawrence, 2014, 2015; van Kampenhout et al., 2017), plant hydraulic stress (Kennedy et al., 2019), and the capacity to simulate transient land use that includes a prognostic crop model (Lawrence et al., 2019). While extending the scientific capacity of CLM, these modifications also improved model performance relative to the ILAMB benchmarking metrics (Lawrence et al., 2019). Here we briefly summarize significant developments in the biogeochemical representations applied in the latest version of the CLM, with more details available in associated CLM5 papers (Bonan et al., 2019).

The representation of CLM's nitrogen cycle, particularly plant nitrogen dynamics, changed in several major ways as the model developed from versions 4.0 to 4.5 and 5.0. Most importantly, CLM4 and 4.5 calculated instantaneous (or potential) gross primary productivity (GPP), without consideration of soil nitrogen availability (Thornton et al., 2007). Subsequently, N availability and stoichiometric constraints downregulated *potential* GPP to provide *actual* GPP estimates. This approach decouples leaf exchange of carbon from associated water and energy fluxes (Zaehle et al., 2014). Further, CLM4 and 4.5 prescribed foliar nitrogen concentrations (leaf C:N ratios) and associated photosynthetic capacities that were static with regard to environmental conditions. Assumptions in CLM4 and 4.5 that related nitrogen availability to plant productivity were not consistent with observations, especially regarding how plants respond to environmental change. Modifications to CLM5 changed these assumptions in three important ways, with the addition of three new modules.

First, recognizing that leaf nitrogen content is dynamic, implementation of “FlexCN” functionality affords flexible plant stoichiometry and thus a single realized GPP calculation, as opposed to separating potential and actual fluxes (Ghimire et al., 2016). Second, recognizing that foliar photosynthetic capacity responds to environmental conditions and changes over space and time, the Leaf Utilization of Nitrogen for Assimilation (LUNA) module provides a prognostic optimization of maximum leaf carboxylation and electron transport rates (Ali et al., 2016; Xu et al., 2012; see also Ellsworth et al., 2004; Kattge et al., 2009; Walker, Beckerman, et al., 2014). Third, recognizing that changes in nitrogen supply or demand have consequences for plant productivity, the Fixation and Uptake of Nitrogen (FUN) module calculates the carbon costs of various nitrogen acquisition strategies and adjusts carbon expenditure on nitrogen uptake among biological fixation, active uptake, and retranslocation (Brzostek et al., 2014; J. B. Fisher, Sitch, et al., 2010; Shi et al., 2016; see also Rastetter et al., 2001). Note, as currently implemented the plant C costs calculated by FUN only serve as an additional source of autotrophic respiration, not as an actual belowground C flux to roots or mycorrhizae that interacts with the soil biogeochemistry simulated in CLM5. Collectively, FlexCN, LUNA, and FUN more mechanistically represent plant nitrogen dynamics in CLM5 and afford opportunities to understand, refine, and improve simulated C‐N biogeochemical dynamics. In addition to these modifications to plant nitrogen cycling, the dynamic plant carbon allocation scheme that was used in CLM4 and 4.5 produced biased plant productivity‐biomass relationships, at least in the tropics (Negrón‐Juárez et al., 2015); thus, a fixed allocation scheme, which assigns carbon in constant proportions to leaves, stems, and coarse and fine roots, was applied in CLM5. Modifications to plant allocation have indirect influences on nitrogen dynamics by altering the nitrogen requirements for a given level of net primary productivity (NPP).

### 2.2 Simulations and Analyses

The simulations presented here were initialized using the baseline (control) simulations presented in Lawrence et al. (2019). Briefly, simulations of CLM4, 4.5, and 5 were spun up to bring the carbon pools to equilibrium by cycling over the first 20 years of the GSWP3v1 climate forcing data set (http://hydro.iis.u-tokyo. ac.jp/GSWP3/). Subsequently, all versions of the model were run through the historical period (1850–2014) at ~1° horizontal spatial resolution using fully transient atmospheric CO_2_ concentrations, nitrogen deposition rates, and land use change data sets that are consistent with second‐generation land use harmonization and CMIP6 protocols (Lawrence et al., 2016; Lawrence et al., 2019). In these control simulations we focused on differences in the spatial distribution of GPP among different versions of the model (averaged over 2010–2014). We compare results with globally gridded GPP estimates from FLUXNET‐MTE (Beer et al., 2010; Jung et al., 2011), and calculated the bias from CLM simulations and FLUXNET‐MTE averaged over the periods 1995–2008. In general, changes to GPP tend to cascade through relevant ecosystem fluxes, like NPP, and pools, like vegetation carbon, and soil carbon stocks. Thus, we also calculated area weighted global sums of relevant ecosystem fluxes and pools.

For this study, we started two additional runs in 1995 where we initiated a global step increase of (1) nitrogen deposition—5 g N m^−2^ yr^−1^ above ambient (evenly distributed over the year through the N deposition stream) or (2) atmosphere CO_2_ concentration—200 ppm over ambient and ran the model forward until 2014. Although individual experimental manipulation used different amounts of N or CO_2_ enrichment, global values used in our simulations were chosen for N enrichment in order to remain on the low side of N‐addition experiments (Liu & Greaver, 2010) and the upper limit of projected N deposition rates over the twenty‐first century (Frey et al., 2014; Lamarque et al., 2013). Similarly, values for CO_2_ enrichment were chosen to approximate experimental manipulations for Free Air CO_2_ Enrichment (FACE) experiments (Ainsworth & Long, 2005).

To quantify model sensitivities to nitrogen enrichment and elevated CO_2,_ we compared the effect size (treatment/control) averaged for each grid cell over the last five years of the simulation (2010–2014). Across grid cells, we report the mean effect size and the 50% prediction interval (calculated to provide an estimate of spatial variation) in each version of the model and compare simulated results with available observations from meta‐analyses of experimental manipulations (see below). To avoid biasing global results with grid cells having small initial carbon fluxes (and thus potentially very large effect sizes), we only calculated effect sizes where mean annual GPP was greater than 100 g C m^−2^ yr^−1^. Where appropriate, we repeated the analysis for each plant functional type (PFT) within grid cells and weighted mean results by the fractional area of each PFT. The broadleaf evergreen shrub PFT represents a small fraction of the vegetated land area in these simulations (<1,000 km^2^) and was excluded from PFT‐level analyses.

Several meta‐analyses quantify aspects of ecosystem responses to nitrogen enrichment. In this study, we compared simulated NPP responses to nitrogen enrichment to the aboveground net primary productivity response reported by LeBauer and Treseder (2008); simulated effect sizes for NPP and aboveground net primary productivity are nearly identical (Wieder unpublished). We compared changes in simulated vegetation carbon stocks with observed changes in total tree biomass in response to N enrichment reported by Janssens et al. (2010). We also compared changes in litter and soil carbon pools with observations reported by Lu et al. (2011) (see also Liu & Greaver, 2010, who report similar results, but with fewer sites). Finally, heterotrophic respiration rates simulated by CLM in response to nitrogen enrichment were compared with observations from Janssens et al. (2010).

Plant physiological and ecosystem responses to elevated CO_2_ simulated by CLM were compared with results synthesized in a meta‐analysis by Ainsworth and Long (2005). Some meta‐analyses from experimental manipulations report the natural log of the response ratio plus or minus the 95% confidence interval (e.g., Ainsworth & Long, 2005). For consistency we back transformed these values and report the observed mean effect size and 95% confidence interval. We recognize a temporal mismatch between the length of experimental manipulations (generally less than a decade) and their spatial bias (toward temperate ecosystems) with the multidecade and global‐scale simulations used in this analysis. We also acknowledge the mismatch in scales between leaf photosynthetic assimilation that can be measured in experimental manipulations and the upscaled canopy GPP that is simulated in the model. Despite these scale mismatches, we contend that the general trends in sign and magnitude of effect sizes that observed in experimental manipulations are still useful to compare to model results. Finally, we recognize that other syntheses of experimental manipulations have been published, and we use some of these studies to contextualize our main findings in the discussion of results.

## 3 Results

### 3.1 Control Simulations

**Figure 1:**
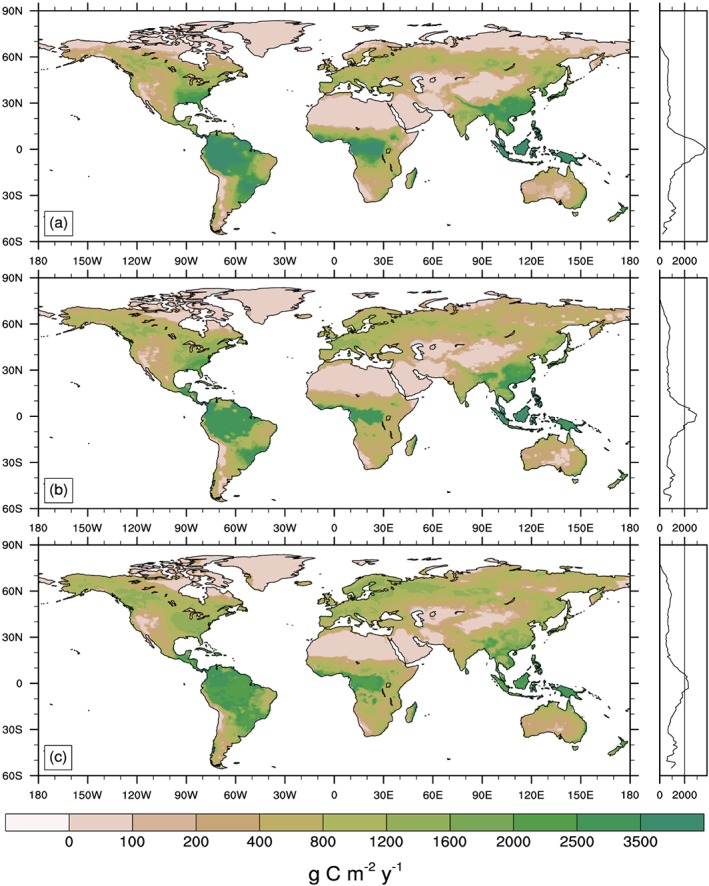
Spatial distribution and zonal mean plots of mean annual gross primary productivity (GPP) simulated in successive versions of the Community Land Model (a) CLM4, (b) CLM4.5, and (c) CLM5 under common atmospheric forcings from GSWP3 (control simulation). All units are g C m^−2^ yr^−1^ and averaged over the last five years of the control simulation (2010–2014).

**Figure 2:**
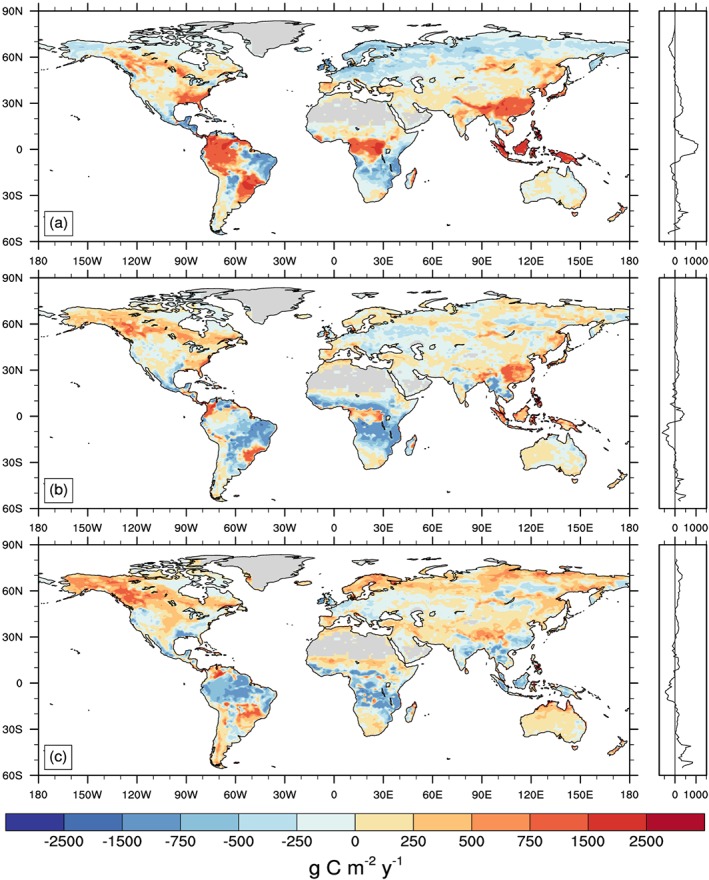
Spatial distribution and zonal mean plots showing biases in mean annual GPP simulated by successive versions of the Community Land Model (a) CLM4, (b) CLM4.5, and (c) CLM5 compared to observationally derived estimates from FLUXNET‐MTE. All units are g C m^−2^ yr^−1^ and averaged over the period 1995–2008.

The three different versions of CLM showed large differences in the spatial distribution and global sums of relevant biogeochemical fluxes and stocks (Table 1). For example, GPP—the gateway through which CO_2_ enters terrestrial ecosystems—simulated by CLM4 was very high in tropical forests and low across the Arctic, compared to later versions of the model (Figure 1). Reducing biases in GPP, relative to FLUXNET‐MTE observations (Figure 2a), was a major focus of subsequent model development (Bonan et al., 2011; Bonan et al., 2012; Koven et al., 2013; Lawrence et al., 2019). Successive versions of the model subsequently showed an overall reduction in global GPP (Table 1), with a shift in the spatial distribution of GPP from the tropics to higher‐latitude ecosystems (Figures 1b and 1c). This reduced the global GPP bias from +12.1, −4.6, to +1.0 Pg C yr‐1 in versions 4, 4.5, and 5 of the model, respectively (Figure 2). Significant biases in GPP remain in CLM5, notably where modeled GPP is too high across high latitudes (Figure 2c); but on balance, newer versions of the model appear to more realistically capture historical spatial patterns and the total amount of terrestrial productivity.

**Table 1:** Global Sums of Ecosystem Fluxes and Stocks and Their Change Relative to the Control Run After Nitrogen and CO_2_ Enrichment Simulated by Successive Versions of the Community Land Model

Model	Treatment	GPP (Pg C yr‐1)	NPP (Pg C yr‐1)	Veg C (Pg C)	Veg N (Pg N)	Soil C (Pg C)	Soil N (Pg N)	N fix (Tg N yr‐1)
	Control	132.9	45.6	472	3.0	513	50.3	109
4	+N	41.4	17.2	67	0.9	44	4.1	24
	+CO_2_	10.1	2.9	28	0.2	1	0.1	3
	Control	116.4	48.2	469	3.2	1027	91.0	98
4.5	+N	24.6	13.9	34	0.6	35	3.1	9
	+CO_2_	7.8	3.0	30	0.2	8	0.3	3
	Control	122.2	50.5	500	4.7	1100	97.6	99
5	+N	12.1	11.3	22	0.5	35	3.0	−35
	+CO_2_	22.6	9.2	65	0.4	14	1.0	53

All sums for gross primary productivity (GPP), net primary productivity (NPP), vegetation carbon and nitrogen stocks (Veg C and N), soil organic matter stocks (Soil C and N, which includes soil and litter stocks, 0–100 cm for CLM4.5 and CLM5), and nitrogen fixation (N fix, the sum of free‐living and symbiotic N fixation in CLM5) are averaged over the last five years of simulations (2010–2014) that were forced with GSWP3 climate reanalysis.

Despite overall reductions in GPP between CLM4 and CLM5, net primary productivity (NPP), or the amount of carbon retained in ecosystems to build plant biomass, slightly increased with successive iterations of the model (Table 1). As with GPP, NPP increased at high latitudes and declined in the tropics in CLM4.5 and 5, relative to CLM4. Globally averaged ecosystem carbon use efficiency (calculated here as the ratio of NPP to GPP) increased from 0.34 in CLM4 to 0.41 in versions 4.5 and 5 of the model (Figure S1). For comparison, observations suggest that efficiencies range from 0.4 and 0.6 across multiple ecosystems, with higher values in managed and more fertile ecosystems (Campioli et al., 2015; DeLucia et al., 2007; Vicca et al., 2012). In tropical forests CLM5 estimates carbon use efficiency values between 0.3 and 0.45 (Figure S1), which also compare favorably with observational estimates between 0.32 and 0.49 by Malhi et al. (2009). Thus, CLM5 seems to simulate more realistic carbon use efficiency values across most ecosystems than previous versions of the model, perhaps with the exception of tropical savannas—although data from these ecosystems are sparse.

The allocation of NPP to vegetation carbon pools with different turnover times, and subsequent transfers to litter and soil C pools, resulted in variation in vegetation and soil C stocks. Global vegetation C stocks varied by 31 Pg C among models, roughly 6% (Table 1), but these totals mask important geographic and ecological variation among models. As with plant productivity, CLM4.5 and 5 both show a poleward shift in vegetation C stocks, relative to estimates in CLM4. Vegetation C stocks averaged over evergreen tropical forests decreased in successive versions of the model (from 25, 24, and 20 kg C m^−2^ in CLM4, 4.5, and 5, respectively), and increased in boreal needleleaf evergreen forests (from 3.9, 6.5, and 16 kg C m^−2^, respectively). Similar patterns are evident in soil C stocks, where CLM4 maintained notably low belowground C stocks (Table 1). Shifting to the CENTURY‐like representation of vertically resolved soil biogeochemistry in CLM4.5 (Koven et al., 2013), combined with higher productivity in high‐latitude ecosystems, more than doubled belowground C stocks relative to CLM4 (data for CLM4.5 and 5 are for top meter of soil). As noted earlier, the terrestrial C stocks and fluxes simulated by CLM5 are improved, relative to previous versions of the model and according to the ILAMB benchmarking package (Lawrence et al., 2019).

### 3.2 Nitrogen Fertilization

**Figure 3:**
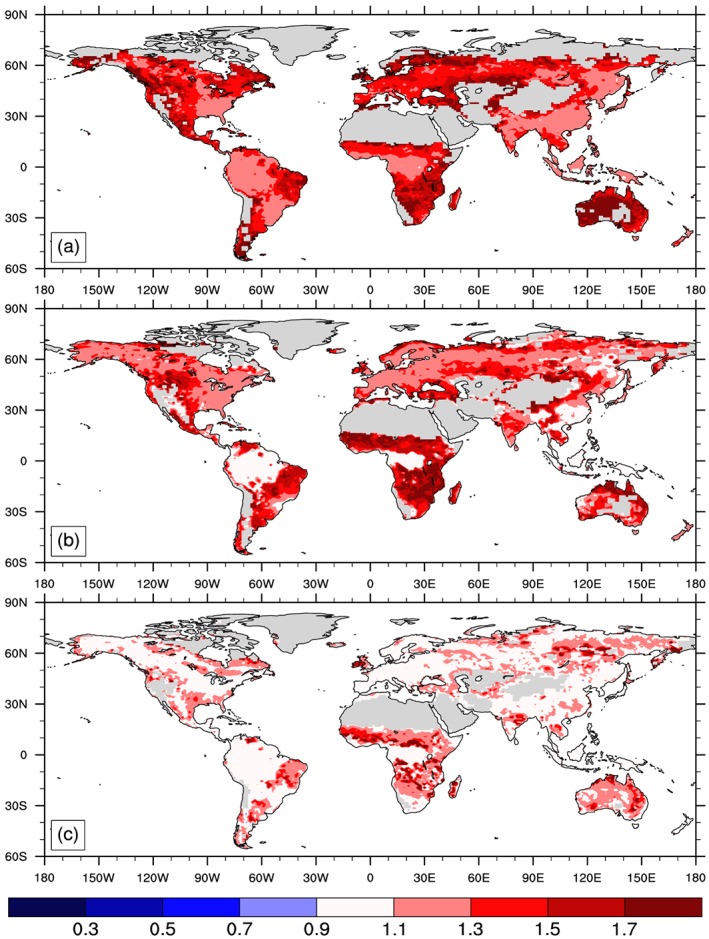
Spatial distribution and zonal mean plots showing the effect size of nitrogen enrichment on GPP simulated by successive versions of the Community Land Model (a) CLM4, (b) CLM4.5, and (c) CLM5. Effect sizes were calculated for each grid cell as the mean annual GPP of treatment divided control simulations over the last five years of the experiment (2010–2014) using cells with mean GPP > 100 g C m^−2^ yr^−1^.

**Figure 4:**
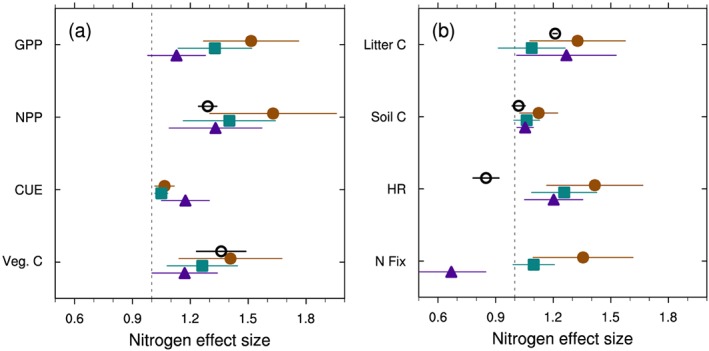
Observed (open circles) and simulated (solid shapes) effect size of nitrogen enrichment on select ecosystem fluxes and pools. Observations, where available, show the mean (±95% confidence interval) from various meta‐analyses (see section 2). Modeled responses show the global mean of grid cell effect sizes (±50% prediction interval) for version 4, 4.5, and 5 CLM (brown circles, turquoise squares, and purple triangles, respectively), calculated using cells with mean GPP > 100 g C m^−2^ yr^−1^. The vertical dashed line represents no effect. Variables listed include (a) gross and net primary productivity (GPP and NPP), carbon use efficiency (CUE), vegetation carbon pools (Veg C) and (b) litter and soil C pools, heterotrophic respiration (HR), and N fixation (N fix) rates.

Nitrogen fertilization increased GPP in all models, but this effect was strongest in CLM4 where global GPP increased 31% relative to the control simulation, compared to a 21% increase in CLM4.5 and to a 10% increase with CLM5 (mean of 2010–2014 values; Table 1). Across biomes, the nitrogen effect size for GPP was also largest for CLM4 and smallest for CLM5 (Figure 3). Variation in the nitrogen effect size among PFTs with CLM5, however, was greater than the average differences across versions of CLM (Table S1). The dampening in N effect size with successive model versions also generally held for multiple carbon pools and fluxes downstream of gross primary productivity (Figure 4). At the grid scale level, CLM4 showed a 60% increase in NPP in response to N enrichment, almost double the 29% increase in aboveground NPP reported in observations (Figure 4a; LeBauer & Treseder, 2008). CLM4.5 and 5 better approximated the mean observed response, although considerable variation among individual PFTs remained (Table S1). By contrast, observed changes in vegetation C stocks (Janssens et al., 2010) were better approximated in CLM4, while CLM5 underestimates changes in vegetation C storage under N fertilization (Figure 4). We note that observed changes in vegetation C stocks shown in Figure 4a, however, mostly come from very young forested ecosystems (Janssens et al., 2010). Yet in the CLM5 simulations, woody PFTs generally showed more muted effect sizes for both NPP and vegetation C stocks compared to observations (Table S1), suggesting that additional modifications are necessary to represent N limitation of forest ecosystems and capture appropriate changes in productivity and allocation that occur in response to N enrichment in woody PFTs. Globally, CLM4.5 and 5 both appear to better approximate observed changes in litter and soil C stocks in response to N fertilization (Figure 4b). When combined with NPP effects, these findings suggest that later versions of the model may better simulate global C sensitivity to N enrichment.

We also present simulated and observed changes in heterotrophic respiration, as well as simulated rates of nitrogen fixation in response to N enrichment (Figure 4b). In the models, increases in productivity result in increased litterfall rates that lead to accumulation of carbon in litter and, ultimately, soil carbon pools (Figure 4). Thus, N fertilization necessarily increases heterotrophic respiration rates in models where decomposition CO_2_ fluxes are directly proportional to upstream C pool sizes (Figure 4b). Observations from experimental manipulations, however, suggest that under some circumstances N enrichment drives shifts in soil microbial community composition and often suppresses decomposition rates and heterotrophic respiration rates (Frey et al., 2014; Janssens et al., 2010; Knorr et al., 2005; Leff et al., 2015; Liu & Greaver, 2010; but see also Lu et al., 2011).

Finally, modeled responses of nitrogen fixation highlight uncertainties about how to represent ecosystem responses to N enrichment. Following N enrichment, N fixation rates increased dramatically with CLM4, but decreased in CLM5 (Figure 4b). This change in sign reflects differences in how N fixation was simulated. In CLM4 and 4.5, nitrogen fixation was estimated using an empirical relationship between N fixation rate and NPP (Cleveland et al., 1999; Wieder, Cleveland, Lawrence, & Bonan, 2015). As such, fertilization‐driven increases in modeled NPP subsequently drove increases in estimated N fixation rates. In CLM5, declines in N fixation with fertilization reflect decreases in plant N uptake costs calculated by FUN relative to the energetic cost for N fixation. In both natural and agricultural systems nitrogen enrichment reduces rates of biological N fixation (Batterman et al., 2013; McAuliffe et al., 1958), suggesting that the decreases in N fixation rates simulated by FUN in CLM5 are likely more appropriate. These contrasting model responses more broadly highlight uncertainties among models in how to appropriately represent ecological processes like fixation in global‐scale models (Meyerholt et al., 2016; Wieder, Cleveland, Lawrence, & Bonan, 2015). Overall, structural modifications to CLM5 muted the global carbon cycle response to nitrogen fertilization.

### 3.3 Elevated CO_2_

**Figure 5:**
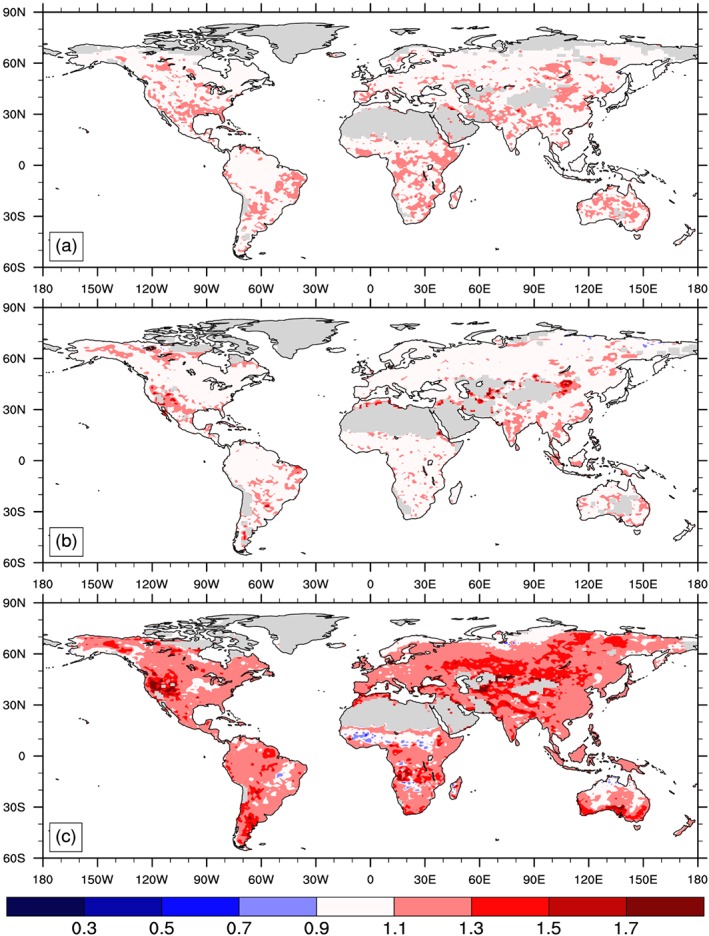
Spatial distribution and zonal mean plots showing the effect size of CO_2_ enrichment on GPP simulated by successive versions of the Community Land Model (a) CLM4, (b) CLM4.5, and (c) CLM5. Effect sizes were calculated for each grid cell as the mean annual GPP of treatment divided control simulations over the last five years of the experiment (2010–2014) using cells with mean GPP > 100 g C m^−2^ yr^−1^.

Increasing atmospheric CO_2_ concentrations by 200 ppm increased productivity in all three versions of the model, increasing GPP just over 7% in CLM4 and 4.5, but eliciting an 18% increase in GPP in CLM5 relative to the control simulation (Table 1). The low CO_2_ effects in CLM4 and 4.5 were equally distributed across the globe, although deciduous tropical forests and C_3_ nonarctic grasses showed the largest response in productivity and downstream C stocks (Figures 5a and 5b and Table S2). By contrast, nearly all PFTs showed significant increases in productivity and downstream ecosystem C stocks in CLM5, with the exception of C_4_ plants (Figure 5c and Table S2). Indeed, regions dominated by C_4_ grasses and C_4_ crops showed negligible effects to elevated CO_2_ in all versions of the model (Table S2), although this pattern is most evident in the CLM5 simulations (Figure 5c). This result aligns well with shorter‐term studies in grasslands and theoretical expectations of the response of the C_4_ photosynthetic pathway to elevated concentrations of CO_2_ (Ainsworth & Long, 2005; Ehleringer & Bjorkman, 1977); however, recent work by Reich et al. (2018) calls into question this established paradigm.

**Figure 6:**
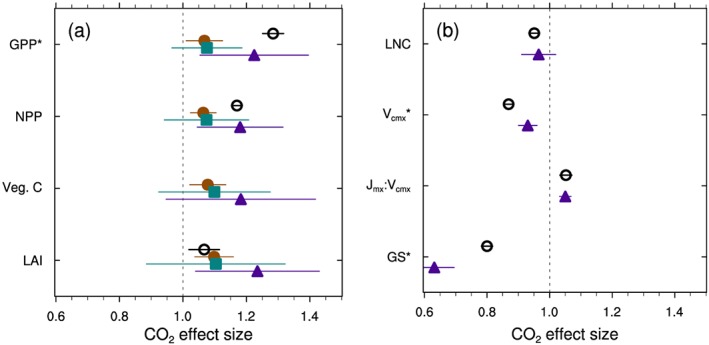
Observed (open circles) and simulated (solid shapes) effect size of CO_2_ enrichment on various ecosystem carbon and leaf traits. Observations, where available, show the mean (±95% confidence interval; Ainsworth & Long, 2005). Modeled responses show the global mean of grid cell effect sizes (±50% prediction interval) for versions 4, 4.5, and 5 of CLM (brown circles, turquoise squares, and purple triangles, respectively), calculated using cells with mean GPP > 100 g C m^−2^ yr^−1^. The vertical dashed line represents no effect. Variables listed include (a) gross and net primary productivity (GPP and NPP), vegetation carbon pools (Veg C), leaf area index (LAI) and (b) leaf N content (LNC), maximum carboxylation rates (V_cmax_), the ratio of maximum electron transport rates (J_max_) to V_cmax_, and stomatal conductance (GS). Asterisk denotes values for the mean of daily maximum rates.

Canopy‐scale measurement of many relevant fluxes are not commonly made in FACE experiments, since the scale of CO_2_ enrichment plots is smaller than the typical footprint of an eddy covariance tower; therefore, we compared the response ratio of leaf measurements from FACE experiments with their closest canopy‐scale model analogue in Figure 6. For example, Ainsworth and Long (2005) report that diurnal photosynthetic assimilation (*A*′) increased 28% under elevated CO_2_, which compares well to the mean of daily maximum GPP rates simulated by CLM5, but not with previous versions of the model (Figure 6a). Similarly, observed dry matter production increased in the FACE experiments by 17% under elevated CO_2_ (with the largest increases in trees), results which again compare favorably to changes in NPP simulated by CLM5. Changes in NPP under elevated CO_2_ resulted in greater accumulations of vegetation C stocks, which increased 28, 30, and 65 Pg C in successive versions of the model (Table 1). However, the CO_2_ induced increases in productivity and vegetation C storage for CLM5 were potentially amplified by increases in leaf area index (LAI) that were greater than observed responses (Figure 6a).

Both CLM4 and 4.5 applied fixed foliar C:N ratios, but CLM5 includes the capacity for flexible tissue stoichiometry (Ghimire et al., 2016), a feature which predicts declines in leaf nitrogen content (LNC) under elevated CO_2_ of the appropriate sign and magnitude to observed values (Figure 6b). LNC was subsequently used to calculate the maximum rate of carboxylation (V_cmax_) and the maximum rate of electron transport (J_max_), and subsequent rates of photosynthesis, using the LUNA module in CLM5 (Ali et al., 2016). Although simulated V_cmax_ declined under elevated CO_2_, the magnitude of this decrease is less than observations suggest, but changes in the ratio of J_max_ to V_cmax_ were of an appropriate magnitude (Figure 6b). Finally, whereas changes in V_cmax_ simulated in CLM5 are not sensitive enough to elevated CO_2_, the changes in stomatal conductance (g_s_) show a strong sensitivity to CO_2_ enrichment (Figure 6b). This is likely caused by the J_max_:V_cmax_ ratio simulated by the LUNA model (Franks et al., 2017). Observations support reduced stomatal and canopy conductance under elevated CO_2_, but these responses are highly variable and likely less pronounced than the changes simulated by CLM5 (Ainsworth & Long, 2005; Norby & Zak, 2011; Walker, Beckerman, et al., 2014; Walker, Hanson, et al., 2014). Our global simulations indicate that accounting for changes in leaf physiology and stoichiometry in CLM5 increased the model's response to CO_2_ fertilization and better agreement with observations from experimental manipulations.

### 3.4 Trade‐Offs Between N and CO_2_ Limitation

**Figure 7:**
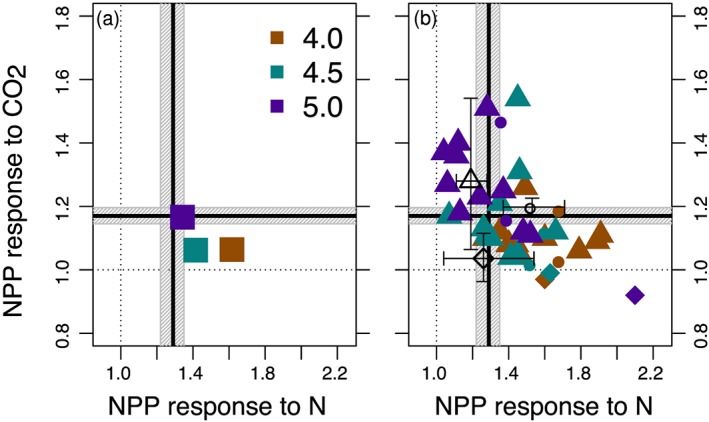
Simulated effect sizes of nitrogen versus CO_2_ enrichment on rates of net primary productivity (NPP) that was calculated (a) globally or (b) for each plant functional type in CLM4, 4.5, and 5 (brown, turquoise, and purple symbols, respectively). In both panels, observational constraints for the nitrogen response (aboveground NPP from LeBauer and Treseder (2008)) and CO_2_ response (dry matter production from Ainsworth and Long (2005)) are shown with the vertical and horizontal lines, respectively (mean ± 95% confidence interval). The right panel shows the observed (open symbols) and simulated (filled symbols) effect sizes of individual plant functional types for woody vegetation, C_3_ grasses, and C_4_ grasses (triangles, circles, and diamonds, respectively).

Figure 7 illustrates broad differences in N and CO_2_ sensitivities among these three versions of the model. We focused on the effect size of NPP to experimental manipulations, as it represents one of the better quantified ecosystem responses and it also serves as a proximal control over the strength of the land C sink simulated in models. Our results illustrate the large sensitivity of terrestrial ecosystems in CLM4 to nitrogen enrichment and their relatively low sensitivity to elevated CO_2_ compared to CLM4.5 and CLM5 (Figure 7a). Adjustments to photosynthesis and soil biogeochemistry in CLM4.5 decreased the N sensitivity so it is closer to measured ranges, but this intermediate model still shows a muted response to elevated CO_2_. At a global scale, CLM5 represents responses to N and CO_2_ enrichment that are more in line with observations, relative to previous versions of the model.

Breaking down these global mean effects into the mean responses of individual plant functional types illustrates the wide variation within and among models (Figure 7b). We highlight several patterns that seem more broadly relevant to the evaluation of terrestrial biogeochemical models. First, observations from experimental manipulations indicate that nutrient availability clearly modulates ecosystem responses to elevated CO_2_ (Norby et al., 2010; Reich et al., 2006; Reich & Hobbie, 2013). Similarly, CLM results suggest that in the model there appears to be a trade‐off between simulated N and CO_2_ responses. That is, a model or PFT that is strongly limited by N availability cannot have a very strong response to elevated CO_2_. Conversely, a model or PFT that shows a strong response to elevated CO_2_ experiences relatively low levels of N limitation. Second, some elements of variation among growth forms appear to be broadly captured by CLM. For example, lower CO_2_ fertilization effects of C_4_ plants are consistent with observations from FACE experiments and captured in the model (Figure 7b and Table S2; Ainsworth & Long, 2005), but contradicted by observations from Reich et al. (2018). The degree of N limitation simulated in C_4_ grasses, however, appears too strong—especially in CLM5, whereas observations suggest that annual C_3_ grasses show the largest response to N enrichment (LeBauer & Treseder, 2008). Woody vegetation shows a greater sensitivity to elevated CO_2_, albeit highly variable, and potentially a lower sensitivity to N (triangles in Figure 7b). Finally, all versions of the model project sustained increases in global‐scale productivity through the 20‐year simulation, a finding not supported by observations from some FACE experiments (Norby et al., 2010; Norby & Zak, 2011). Contradictions between simulated and observed results highlight opportunities to assess, refine, and revise the structural assumptions and particular parameterizations of land models.

### 3.5 Biogeophysical Feedback

Beyond the direct biogeochemical effects of N enrichment and elevated CO_2_, our simulations begin to shed light on potential future biogeophysical feedbacks to water and energy exchange between the land and atmosphere. A motivation for conducting FACE experiments was to assess if reductions in leaf stomatal conductance ultimately influence canopy conductance, evapotranspiration, and runoff—or if concurrent changes in LAI under elevated CO_2_ would attenuate whole plant and ecosystem changes in water use (Norby & Zak, 2011; Wullschleger et al., 2002). In our simulations N and CO_2_ enrichment both increased plant productivity and LAI in all versions of the model, but decreased stomatal conductance was only observed under elevated CO_2_ with CLM5.

The biophysical feedback on latent heat fluxes show similar magnitude but the opposite sign under +N compared to +CO_2_ enrichment. With N addition, average latent fluxes increased 3.6, 2.2, and 2.9% in CLM4, 4.5, and 5, respectively, and decreased global runoff by a similar magnitude. These global similarities, however, mask distinct regional differences among models. For example, CLM4 showed larger changes in latent heat fluxes with N fertilization than other versions of the model, especially in tropical forests (Figure S2). By contrast, nitrogen effects on latent heat fluxes in CLM4.5 and 5 were most prominent in tropical savannas. Under elevated CO_2_, globally averaged latent heat fluxes decreased in all models, by 3.9, 2.8, and 3.5% in CLM4, 4.5, and 5, respectively, which similarly increased global runoff. Again, decreases in latent fluxes under elevated CO_2_ were largest in forested regions with CLM4 and CLM4.5, albeit to a lesser extent, whereas CLM5 shows relatively larger effect in the tropics and in the high latitudes (Figure S3).

## 4 Discussion

Structural and parametric changes in successive versions of the Community Land Model resulted in shifts of terrestrial productivity and carbon storage from tropical forests toward temperate and high‐latitude regions (Figure 1). This reduced GPP bias, relative to estimates from FLUXNET‐MTE (Figure 2; Beer et al., 2010; Jung et al., 2011) and resulted in an overall improvement in model skill score across multiple metrics used to benchmark land models (Lawrence et al., 2019). A number of aspects in the model changed during CLM5 development, as detailed by a companion paper that is part of the CESM2 special issue (Lawrence et al., 2019). We have not isolated individual contributions from particular development activities, but other work begins to quantify sensitivities in parameter perturbation experiments (Fisher et al., 2019), explicit representation of crop management and phenology, and atmospheric forcing uncertainty (Bonan et al., 2019). In all of these efforts the ILAMB model benchmarking package provided critical insights into areas of model improvements and degradation (Collier et al., 2018). Moving forward, we contend that simultaneously assessing the representation of multiple ecological processes in land models will help reduce uncertainty and evaluate the likelihood of future projections (Medlyn et al., 2015). By comparing observed and simulated ecosystem responses to experimental manipulation of ecosystem N and CO_2_ availability we evaluated three versions of the Community Land Model. Over the course of model development, CLM transitioned from a model that showed strong sensitivity to nitrogen enrichment and little response to CO_2_ (in CLM4) to a model with stronger sensitivity to elevated CO_2_ (in CLM5; Figures 3 and 5). On balance, our results suggest that the globally integrated ecosystem responses to nitrogen and CO_2_ enrichment are better approximated with CLM5 than with previous versions of CLM (Figures 4 and 6). These findings are consistent with recent observations suggesting relatively weak N limitations on CO_2_ fertilization of plant productivity over the twentieth century (Campbell et al., 2017). Our results also highlight areas where further work, especially related to changes in plant allocation, is needed to align model assumptions with real‐world ecosystem responses to environmental change.

More broadly, our findings suggest a trade‐off between nitrogen and carbon limitation in land models. This trade‐off can be seen both among different versions of the CLM and between individual plant functional types represented in the model (Figure 7). Globally, averaged responses to nitrogen and CO_2_ enrichment suggest that successive versions of the model are better at approximating plant productivity responses to these experimental manipulations (Figure 7a). Individual PFT responses suggest that particular assumptions and parameterizations across versions of CLM tend to favor either N or C limitation of plant productivity (Figure 7b). Evaluating whether similar trade‐offs occur in natural ecosystems will help improve the representation of coupled biogeochemical cycles in land models. For example, recent studies suggest that plant nutrient economies largely determine ecosystem responses to elevated CO_2_, but that long‐term shifts in belowground function and nutrient cycling may be slow to manifest (Reich et al., 2018; Terrer et al., 2018). Our findings also suggest that greater attention should be given to model assumptions and ecosystem traits related to plant N acquisition strategies and the nature of plant‐soil feedback (Fisher et al., 2019; Rastetter et al., 1997). More widely testing these findings may be helpful in disentangling uncertainties in upcoming model intercomparison projects (e.g., C4MIP or LUMIP; Jones et al., 2016; Lawrence et al., 2016), with the aim of ultimately improving global‐scale terrestrial biogeochemical projections. Below we discuss the model assumptions that are responsible for simulated sensitivities to nitrogen and CO_2_ enrichment and highlight avenues for future research related to plant allocation and plant‐soil interactions.

### 4.1 Nitrogen Fertilization

Uncertainties in how to represent nitrogen effects on photosynthetic carbon uptake lead to divergent model responses to nitrogen enrichment (Meyerholt & Zaehle, 2015; Thomas, Bonan, & Goodale, 2013; Table 1 and Figure 3). The separate calculation of potential and nitrogen‐downregulated GPP in CLM4 and 4.5 generates the large GPP responses to N fertilization (Figures 3a and 3b; Thomas, Zaehle, et al., 2013). It is possible to reduce the N fertilization response within this approach by decreasing parameter values for the maximum photosynthetic rate. In particular, in CLM4.5, Bonan et al. (2011, 2012) decreased the potential photosynthetic rates of evergreen tropical trees to more realistic levels which accounts for the lower sensitivity of tropical forests to N enrichment, relative to CLM4 simulations (Figure 3). The FlexCN module in CLM5 removes the assumption of N unlimited potential GPP, instead calculating plant photosynthetic capacity as a function of time‐varying foliar N content (Fisher et al., 2019; Ghimire et al., 2016). Thus, changes in GPP with N fertilization are more dampened in CLM5 (Figure 3c), and attributable to measurable changes in ecosystem properties like leaf nitrogen content and LAI.

Nitrogen enrichment increased LNC in CLM5 by only 3% globally whereas LAI increased 25%. Observations, however, indicate that nitrogen enrichment typically drives relatively larger changes in LNC and more modest changes in leaf biomass (Xia & Wan, 2008). Additional experimental manipulations and meta‐analyses may be warranted here, as current work tends to bias sampling in late successional or closed canopy ecosystems where changes in LNC may be more apparent. Our results, however, indicate that additional model developments may be necessary to capture the correct sensitivities of both LNC and LAI to nitrogen availability. Such concerns are not trivial, as they involve reconciling assumptions and interactions between plant allocation, flexible stoichiometry represented by FlexCN, and C costs of N uptake that are calculated by FUN. Both FlexCN and FUN are new, independently developed contributions for CLM5 that address particular gaps in the model, but reconciling how these modules interact with each other as well as the plant allocation scheme and competition for soil N remains a challenge. Observations from experimental manipulations consistently show shifting patterns of plant C and N allocation (Janssens et al., 2010; Liu & Greaver, 2010) and provide a robust set of targets that models should try to replicate.

Our results highlight model assumptions that generate distinct ecosystem responses to nitrogen enrichment among versions of CLM. For example, CLM5 shows a stronger NPP response to N enrichment, relative to GPP, which increases plant carbon use efficiency (Figure 4a). Increasing carbon use efficiency in CLM5 results because the FUN module calculates lower carbon costs of nitrogen uptake following N enrichment (Fisher et al., 2019; Shi et al., 2016). This allows plants to build more biomass per unit of GPP. Further, the greater ecosystem N availability also decreases rates of symbiotic nitrogen fixation since active uptake of soil N is cheaper than N fixation, and thus preferred by plants (Table 1 and Figure 4). Indeed, observations do suggest that fertile ecosystems build biomass more efficiently, mainly through shifting allocation of C to aboveground biomass (Campioli et al., 2015; Vicca et al., 2012), and that nutrient enrichment reduces biological nitrogen fixation rates (Batterman et al., 2013; McAuliffe et al., 1958). Thus, assumptions in the FUN module that adjust plant C expenditure on nitrogen acquisition and sources of nitrogen uptake are supported by ecosystem observations. Other assumptions, however, related to heterotrophic respiration fluxes and shifts in plant C allocation (see section 4.3) are not well supported by data.

Heterotrophic respiration fluxes simulated in models that apply first‐order soil biogeochemical models are directly proportional to litter inputs and soil carbon stocks (see Gerber et al., 2010). Thus, increasing productivity in response to nitrogen enrichment necessarily increases heterotrophic respiration fluxes (Figure 4b). Meta‐analyses, however, show weakly positive (Lu et al., 2011) to strongly negative responses of heterotrophic respiration and litter decomposition to N enrichment (Janssens et al., 2010; Knorr et al., 2005; Liu & Greaver, 2010). Indeed, shifting plant allocation away from belowground investment for nutrient acquisition toward production of aboveground biomass may directly reduce root and heterotrophic respiration rates, especially in rhizosphere soils and in ecosystems where plants associate with ectomycorrhizal fungi (Phillips et al., 2013; Phillips & Fahey, 2007; Treseder, 2004). None of the three versions of CLM appropriately capture observed shifts in plant C allocation and belowground ecosystem function in response to nitrogen enrichment, in part because they do not yet consider plant allocation of carbon to microbial priming or mycorrhizae. These discrepancies highlight assumptions in the model that deserve further revisions, especially regarding how ecosystems respond to changes in resource availability.

### 4.2 Elevated CO_2_

The low sensitivity of CLM4 to elevated CO_2_ concentrations has been noted elsewhere (Koven et al., 2013; Zaehle et al., 2014), leading to biases in the atmospheric fraction of anthropogenic CO_2_ emissions in coupled simulations (Hoffman et al., 2014). Similar patterns are clearly evident in the global results presented here (Figure 5a). Given modifications to CLM4.5 that resulted in improvements in the terrestrial C balance over the twentieth century (Koven et al., 2013), we expected this intermediate version of the model to have a stronger sensitivity to elevated CO_2_. Our results, however, contradict this expectation, with effect sizes for GPP to elevated CO_2_ in CLM4.5 that largely mirror those from CLM4 (Figure 5b and Table 1). In contrast, CLM5 simulates larger increases in GPP with CO_2_ enrichment (Figure 5c), and the magnitude of change for both GPP and NPP simulated by CLM5 seem to better match observations (Figure 6a). We note that short‐term increases in leaf photosynthesis in response to CO_2_ enrichment have been reported elsewhere, but that concurrent changes in aboveground NPP are not universally observed—especially over longer time scales (Ellsworth et al., 2017; Norby et al., 2005; Norby et al., 2010). Thus, both CLM4.5 and CLM5 are able to match globally integrated estimates of the land carbon sink over the historical period, but by different means (Bonan et al., 2019; Koven et al., 2013; Lawrence et al., 2019).

Developments in CLM5 provide insights into the assumptions responsible for model responses to elevated CO_2_. For example, CLM5 applies a prognostic calculation of foliar nitrogen content and photosynthetic capacity, which were fixed parameters for each PFT in CLM4 and 4.5 (Ali et al., 2016; Fisher et al., 2019; Ghimire et al., 2016). Under elevated CO_2_, foliar nitrogen concentrations decrease by an appropriate magnitude, compared to observations from FACE experiments (Figure 6b; Ainsworth & Long, 2005). As expected, V_cmax_ calculated by the LUNA module and implemented in CLM5 decreased, but it still remained too high compared to observations from FACE experiments (Figure 6b; Ainsworth & Long, 2005). Indeed, the relative importance of electron transport, Rubisco, and triose phosphate utilization limited photosynthesis under elevated CO_2_ from observations remains uncertain (Franks et al., 2017; Lombardozzi et al., 2018; Medlyn et al., 2015). Thus, future efforts should consider the assumptions and parameterizations related to photosynthetic limitation and stomatal conductance in order to better match observations from FACE experiments. By introducing flexible leaf stoichiometry and prognostic photosynthetic capacity CLM5 more faithfully represents leaf physiology, but these advancements also introduce new uncertainties—especially related to leaf photosynthetic and stomatal responses to elevated CO_2_ and their canopy scaling.

Observed changes in vegetation and ecosystem C stocks under elevated CO_2_ remain uncertain, with comparisons among sites complicated by changes in plant C allocation, nutrient availability, and plant‐soil interactions (Norby & Zak, 2011; Reich et al., 2006; Reich et al., 2018; Reich & Hobbie, 2013; Terrer et al., 2018). Increased productivity and vegetation C storage for CLM5, however, were in part driven by increases in LAI that exceeded observed responses (Figure 6a; Ainsworth & Long, 2005). Some of the largest changes occurred in tropical forests (data not shown); and although free air CO_2_ enrichment studies in the Amazon are just beginning (Norby et al., 2015), meta‐analyses from temperate sites suggest that the response of LAI to elevated CO_2_ decreases with canopy closure (Norby & Zak, 2011). Instead, observations show that leaf mass per area (LMA) typically increases under elevated CO_2_, resulting in increased canopy biomass but not LAI (Ainsworth & Long, 2005; Kovenock & Swann, 2018; Medlyn et al., 2015). By contrast, models, including all three versions of CLM presented here, hold LMA at the top of the canopy constant while at the same time they assume that there is a negative LMA profile from the top to the base of the canopy (Thornton et al., 2007; see also Kovenock & Swann, 2018). This means that in the model, the additional increment of leaf biomass under elevated CO_2_ causes the canopy‐mean LMA to actually decrease slightly. It also means that the LAI increases slightly more than the leaf biomass. Thus, with high photosynthetic capacity and greater LAI, the increases in plant productivity and biomass simulated by CLM5 under elevated CO_2_ may be overestimated, especially over multidecadal time scales.

### 4.3 Allocation and Nutrient Uptake

Here we focus on the representation of plant allocation and the temporal dynamics of nutrient limitation, as these topics are relevant to both N and CO_2_ enrichment simulations presented here. The allocation of carbon into different vegetation components (leaves, wood, and fine roots) remains a significant source of uncertainty in land models (Litton et al., 2007; Malhi et al., 2011; Negrón‐Juárez et al., 2015). Meta‐analyses of experimental manipulations suggest that allocation changes, stoichiometric change, and acclimation are important aspects of terrestrial ecosystem responses to environmental change (Liu & Greaver, 2010; Luo et al., 2006; Reich et al., 2006). Our simulations, however, indicate that CLM has limited capacity to capture these responses—highlighting areas to be addressed in future model developments.

Observations demonstrate that plants change their allocation strategies in response to resource availability. For example, nitrogen enrichment tends to favor carbon allocation into woody biomass (at least in forests). It also causes a shift toward aboveground productivity, at the expense of belowground carbon investment (Aerts & Chapin, 2000; Janssens et al., 2010; Liu & Greaver, 2010). By contrast, CO_2_ enrichment tends to increase belowground carbon allocation as plants increase investment in nutrient, or nutrient and water, acquisition (Drake et al., 2011; Iversen et al., 2008), and occasionally an increase in allocation to woody biomass (De Kauwe et al., 2014). Indeed, understanding the nuances of changing belowground allocation in response to various environmental change scenarios is an active field of research (Giardina et al., 2005; Terrer et al., 2018; Treseder et al., 2018), and as such capturing these dynamics in models remains an outstanding challenge.

Both CLM4 and 4.5 applied a dynamic allocation scheme that increased allocation to wood with increases in NPP. This approach qualitatively captured the appropriate allocation response under N enrichment, but not elevated CO_2_ (Figures 4 and 6). This assumption also results in large increases in woody biomass, especially in tropical forests with CLM4 (Bonan & Levis, 2010), and causes an unrealistic productivity‐biomass relationship across tropical forests (Negrón‐Juárez et al., 2015). To rectify these shortcomings, CLM5 uses a fixed allocation scheme where the same fractions of carbon are assigned to leaves, stems, and coarse and fine roots irrespective of NPP (Lawrence et al., 2019). Accordingly, CLM5 cannot capture the changes in belowground allocation that have been observed with N or CO_2_ enrichment. Further, relative allocation to leaves is constant in CLM5, and so leaf allocation is not responsive to the economics of leaves in the lower layers of the canopy, which would in reality experience increasing light limitation. Reduction of resource allocation for leaves in negative carbon balance is a feature of the CLM (FATES) model (R. A. Fisher, McDowell, et al., 2010; Fisher et al., 2015). This allows LAI predictions to be responsive to leaf traits and their implications for the plant carbon economy, and will be incorporated into future releases of CLM.

Mounting evidence suggests that flexible allocation of carbon and nitrogen are necessary to capture observed ecosystem responses to both N and CO_2_ enrichment (De Kauwe et al., 2014; Meyerholt & Zaehle, 2015). The introduction of FlexCN in CLM5 provides appropriate changes in leaf nitrogen content with nitrogen fertilization (Xia & Wan, 2008) and CO_2_ enrichment (Ainsworth & Long, 2005; Figure 6b). Over the course of model development, however, only minor adjustments have been made to assumptions regarding plant N uptake and the nature of plant‐microbial N competition. For example, Koven et al. (2013) modified assumptions to decrease denitrification N losses and increase ecosystem nitrogen retention in CLM4.5 and 5 (see also Fan et al., 2015). Several studies, however, document unrealistically rapid plant N uptake in the CLM (Cheng et al., 2018; Thomas, Bonan, & Goodale, 2013). This could be addressed with alternative model structures that better resolve competitive interactions between plants and microbes (Zhu et al., 2017). Additionally, changes to the cost functions in the FUN module (Brzostek et al., 2014; Shi et al., 2016) in CLM5 could modify plant affinity for N and its change over time. More broadly, these findings highlight opportunities to better incorporate ecological understanding into representation of coupled biogeochemical cycles and their representation in global scale models.

Simulated responses to nitrogen enrichment and elevated CO_2_ also reflect different model assumptions related to plant nutrient acquisition strategies between versions of the CLM. Plants apply multiple strategies to acquire nitrogen, which may include symbiotic N_2_ fixation, as well as associations with different types of mycorrhizal fungi (Goodale, 2017; Nave et al., 2013; Phillips et al., 2013; Terrer et al., 2018; Treseder, 2004). In control simulations, the global sum (Table 1) and spatial distribution of contemporary symbiotic nitrogen fixation seems reasonable in CLM5, although these estimates may be high in temperate and boreal forests in all versions of the model (Houlton et al., 2008; Vitousek et al., 2013). Nitrogen fixation declines in CLM5 with nitrogen fertilization, as plants utilize less energetically expensive N uptake pathways (Figure 4). This response aligns with observations from agricultural systems (McAuliffe et al., 1958)—although fewer data are available for natural vegetation, especially in tropical forests and savannas (Batterman et al., 2013)—and suggests that the sign of the nitrogen fixation response to N fertilization seems more appropriate with CLM5 than previous versions of the model.

Under elevated CO_2_, both CLM4 and CLM4.5 have limited ability to increase N acquisition rates. These versions of the model increased stocks of nitrogen deployed in new growth by roughly 50 Tg N globally (~5%) at the end of the elevated CO_2_ experiment. In contrast, the FUN module of CLM5 affords ways for plants to acquire new nitrogen; thus, plant nitrogen accumulation in new growth increased 150 Tg N globally (~15%) with this version of the model. Much of this nitrogen was supplied by existing ecosystem nitrogen pools, although a significant fraction of “new” nitrogen was fixed via symbiotic nitrogen fixation with CLM5, which doubled under elevated CO_2_ (Table 1). Examples of nitrogen fixing strategies can be found in multiple ecosystems (Houlton et al., 2008; Menge et al., 2009), but we doubt their relative abundance (a parameter that determines the fraction of N fixing species in a PFT) and activity under elevated CO_2_ are as high as assumed in the current CLM5 configuration. Moreover, even where N_2_‐fixing strategies are common, FACE experiments do not support the large and sustained increases in N_2_‐fixation rates currently simulated by CLM5 (Hungate et al., 2004; Reich et al., 2006; van Groenigen et al., 2006), which likely avoids progressive N limitation in the model. As currently applied in CLM5, the carbon costs calculated by FUN are incurred as a plant respiration flux, but future modifications to link plant nutrient limitation with shifts in plant carbon allocation or ability to acquire soil nutrients may serve to correct assumptions in the model and its response to environmental change, especially in a CO_2_‐rich world.

## 5 Conclusion

Model benchmarking serves as a useful tool to objectively quantify areas of model improvements and deficiencies. Indeed, a myriad of parametric and structural changes throughout the family of CLM models evaluated here illustrates improvements in gross primary productivity and other carbon cycle metrics simulated in CLM5 (Lawrence et al., 2019). Moreover, using data from experimental manipulations to compare similarly perturbed model simulations affords additional insights into model assumptions. Our results suggest that ecosystem nitrogen limitation was greater than observed in CLM4 and 4.5, likely because of the assumptions related to nitrogen limitation that downregulated GPP for a higher potential state in these versions of the model. Given their strong N limitation, CLM4 and 4.5 show sensitivities to elevated CO_2_ that are lower than observations. Subsequent modifications to CLM5 afforded more appropriate sensitivities to both nitrogen and CO_2_ enrichment. Results from these global‐scale experimental manipulations suggest that new capabilities in CLM5 that improve plant nitrogen uptake and use—namely FlexCN, LUNA, and FUN—are largely responsible for these improvements.

Our results also suggest that the mechanisms by which CLM5 matches these observational targets deserve further refinement and integration, specifically related to shifts in plant allocation and nutrient acquisition strategies under environmental change. As such, additional research is needed to more completely understand, quantify, and simulate changes in plant physiology, allocation, and nutrient uptake that occur under environmental change. Long‐term experimental manipulations consistently show surprising ecosystem responses that are not expected from theoretical expectations or initial observations (Hungate et al., 2004; Melillo et al., 2017; Norby et al., 2010; Reich et al., 2018). Thus, synthesizing results across diverse ecosystems, continuing collection of long‐term data sets from experimental manipulations, and integrating these insights into models are critical to further refine and evaluate the representation of terrestrial ecosystems in land models that show increasingly sophisticated consideration of ecological processes. By comparing the effect size of modeled and observed responses to experimental manipulations, our approach affords opportunities to move beyond static benchmarks that can be used to understand and evaluate ecological processes that are represented in models.

### Model Availability

CLM5.0 is publicly available through the Community Terrestrial System Model (CTSM) git repository (https://github.com/ESCOMP/ctsm).

6

## Author Contribution

W.R.W. conducted the simulations and wrote the manuscript with contributions from all other authors.

## Supporting information

Supporting Information S1

## Data Availability

Data are publicly available at the UCAR/NCAR Climate Data Gateway, doi:10.5065/d6154fwh.
