# Supplementary material for: Beyond Static Benchmarking: Using Experimental Manipulations to Evaluate Land Model Assumptions
Source: Global Biogeochem Cycles. 2019 Oct 28;33(10):1289–309. doi: 10.1029/2018GB006141 (PMC6919943; doi:10.1029/2018GB006141)
Supplement: Supplementary file 1 — Supporting Information S1 [file GBC-33-1289-s001.pdf]

Global Biogeochemical Cycles

Supporting Information for

Beyond static benchmarking: Using experimental manipulations to evaluate land model assumptions

William R. Wieder[1,2,*], David M. Lawrence[1], Rosie A. Fisher[1], Gordon B. Bonan[1], Susan J. Cheng[3], Christine L. Goodale[3], A. Stuart Grandy[4], Charles D. Koven[5], Danica L. Lombardozzi[1], Keith W. Oleson[1], R. Quinn Thomas[6]

1Climate & Global Dynamics Laboratory, National Center for Atmospheric Research, Boulder, CO 80307, USA

2Institute of Arctic and Alpine Research, University of Colorado, Boulder, CO 80309, USA

3Department of Ecology and Evolutionary Biology, Cornell University, Ithaca, NY, USA

4Department of Natural Resources and the Environment, University of New Hampshire, Durham, NH, USA

5Climate and Ecosystem Sciences Division, Lawrence Berkeley National Laboratory, Berkeley, CA, USA

6Department of Forest Resources and Environmental Conservation, Virginia Tech, Blacksburg, VA, USA

*Corresponding author, W.R. Wieder wwieder@ucar.edu, (303) 497-1352, orchid ID 0000-0001-7116-1985

Contents of this file

Figures S1 to S3
Tables S1 to S2

Introduction

Supporting information that additional figure and tables of results from the simulations presented in the paper.

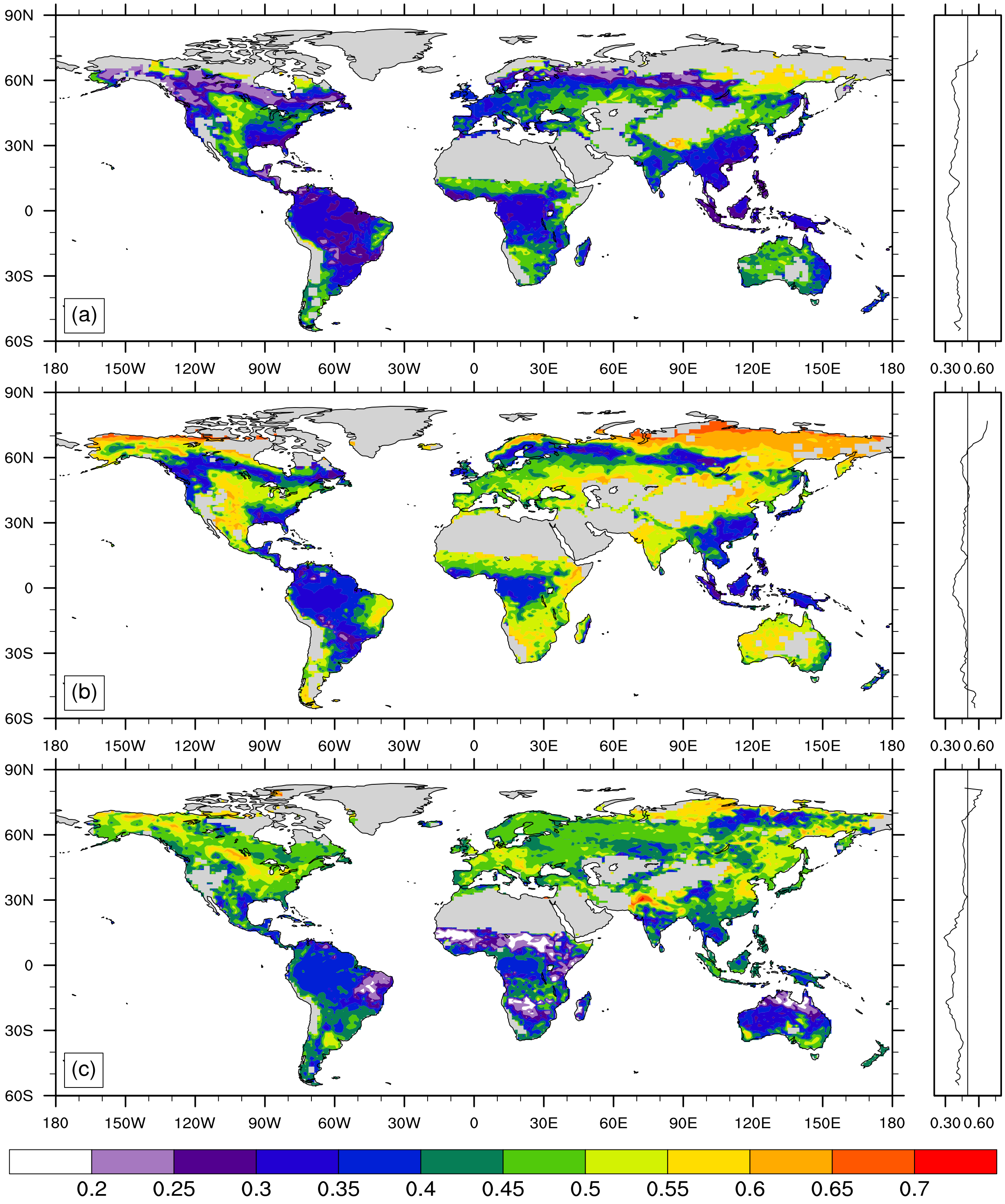

Figure S1. Spatial distribution and zonal mean plots showing ecosystem carbon use efficiency, calculated here as the quotient of mean annual NPP and mean annual GPP simulated by successive versions of the Community Land Model (a) CLM4, (b) CLM4.5, and (c) CLM5. This variable is unitless, and averaged over the last five years of the control simulation (2010-2014).

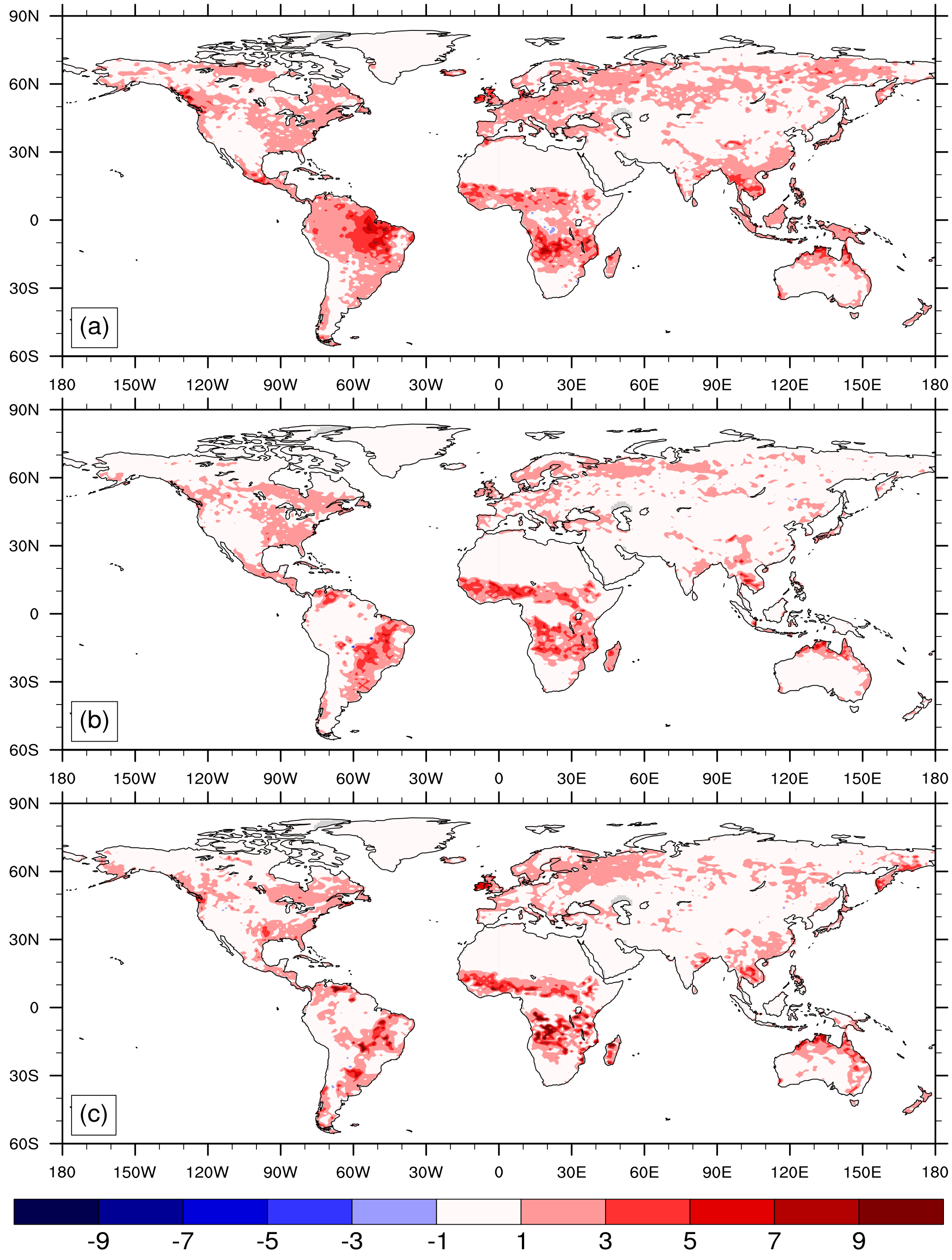

Figure S2. Spatial distribution showing the change in latent heat fluxes (W m^{-2}) in response to nitrogen enrichment simulated by successive versions of the Community Land Model (a) CLM4, (b) CLM4.5, and (c) CLM5. Differences were calculated for each grid cell as the mean annual latent heat flux of treatment minus control simulations averaged over the last five years of the experiment (2010-2014) using cells with mean GPP > 100 g C m^{-2} y^{-1}.

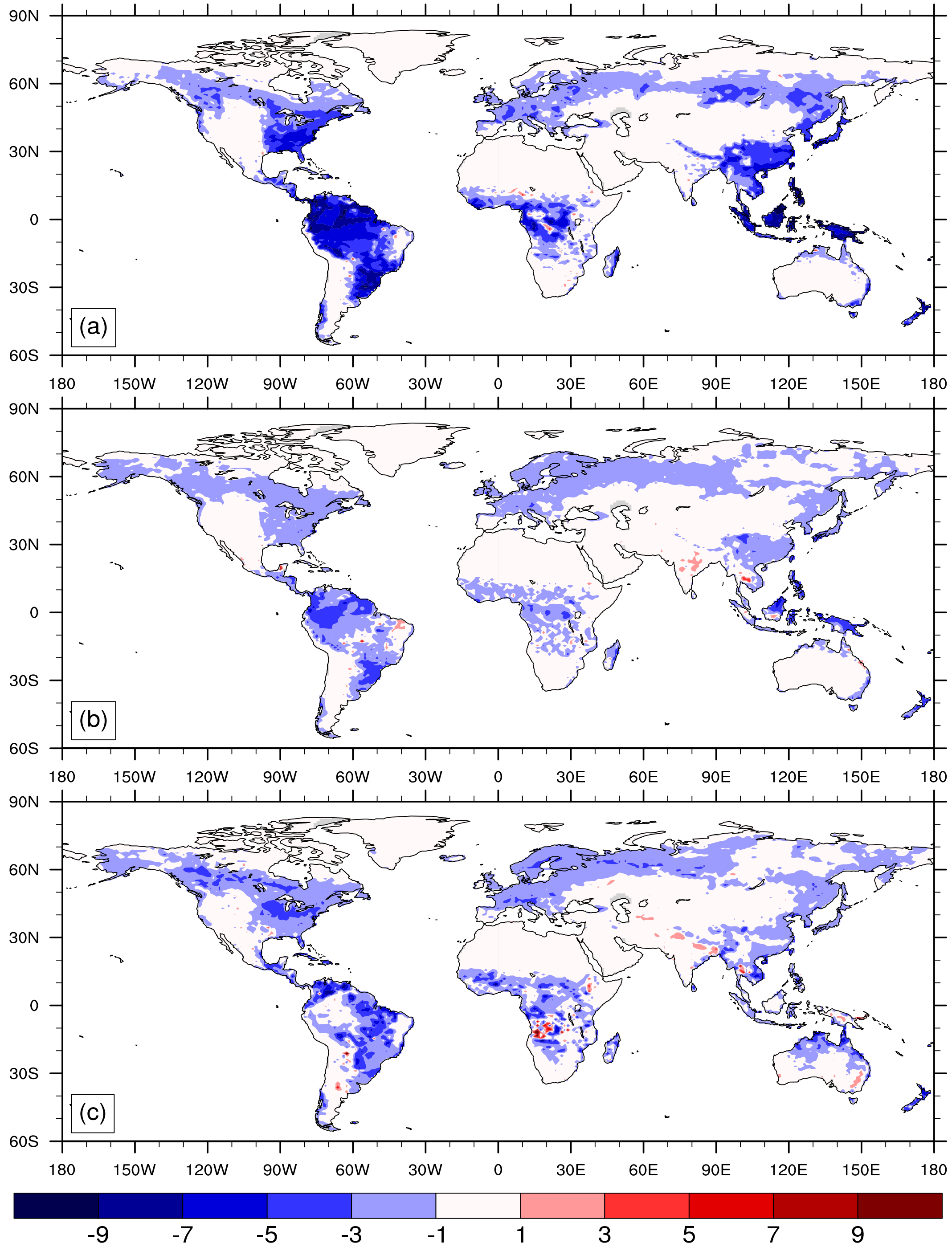

Figure S3. Spatial distribution showing the change in latent heat fluxes (W m^{-2}) in response to CO_2 enrichment simulated by successive versions of the Community Land Model (a) CLM4, (b) CLM4.5, and (c) CLM5. Differences were calculated for each grid cell as the mean annual latent heat flux of treatment minus control simulations averaged over the last five years of the experiment (2010-2014) using cells with mean GPP > 100 g C m^{-2} y^{-1}.

PFT	LAI			GPP			NPP			VEGC		
	4.0	4.5	5.0	4.0	4.5	5.0	4.0	4.5	5.0	4.0	4.5	5.0
NL Evergreen Temperate Tree	1.31	1.18	1.14	1.3	1.18	1.07	1.35	1.26	1.12	1.26	1.15	1.05
NL Evergreen Boreal Tree	1.53	1.18	1.14	1.46	1.18	1.06	1.6	1.28	1.13	1.51	1.16	1.04
NL Deciduous Boreal Tree	1.7	1.91	1.47	1.85	1.71	1.32	1.89	1.66	1.52	1.73	1.57	1.16
BL Evergreen Tropical Tree	1.31	1.06	1.04	1.25	1.06	1.02	1.27	1.07	1.04	1.15	1.06	1.02
BL Evergreen Temperate Tree	1.41	1.3	1.05	1.39	1.31	1.01	1.44	1.35	1.06	1.26	1.33	1.01
BL Deciduous Tropical Tree	1.68	1.43	1.14	1.49	1.46	1	1.49	1.45	1.11	1.41	1.43	1.01
BL Deciduous Temperate Tree	1.36	1.26	1.28	1.36	1.27	1.16	1.4	1.3	1.24	1.28	1.17	1.12
BL Deciduous Boreal Tree	1.44	1.32	1.38	1.45	1.37	1.24	1.47	1.41	1.37	1.3	1.22	1.14
BL Evergreen Shrub	2.51	2.07	1.66	2.36	1.99	1.57	1.51	1.49	1.68	1.31	1.25	1.46
BL Deciduous Temperate Shrub	1.66	1.47	1.25	1.72	1.38	1.11	1.91	1.46	1.28	1.24	1.2	1.11
BL Deciduous Boreal Shrub	1.98	1.45	1.46	1.82	1.43	1.23	1.79	1.45	1.48	1.46	1.23	1.3
C3 arctic grass	2.21	1.53	1.52	1.79	1.51	1.27	1.69	1.53	1.4	1.61	1.52	1.37
C3 non-arctic grass	1.83	1.51	1.38	1.74	1.52	1.2	1.69	1.61	1.37	1.56	1.43	1.27
C4 grass	1.62	1.48	2.38	1.54	1.5	1.48	1.6	1.63	2.1	1.56	1.47	1.78
Global Mean	1.53	1.29	1.27	1.58	1.34	1.14	1.62	1.42	1.34	1.44	1.27	1.17

Table S1 Area weighted effect size (treatment / control) of the N fertilization experiment for each of the natural vegetation plant functional types (PFTs). All values taken from annual means at the end of the experiment (2014). Variables shown here include leaf area index (LAI), gross primary productivity (GPP), net primary productivity (NPP) and total vegetation carbon (VEGC) for each version of CLM (4.0, 4.5, and 5.0).

PFT	LAI			GPP			NPP			VEGC		
	4.0	4.5	5.0	4.0	4.5	5.0	4.0	4.5	5.0	4.0	4.5	5.0
NL Evergreen Temperate Tree	1.13	1.13	1.53	1.13	1.11	1.41	1.12	1.13	1.4	1.13	1.12	1.24
NL Evergreen Boreal Tree	1.09	1.13	1.23	1.08	1.09	1.19	1.1	1.11	1.18	1.08	1.11	1.1
NL Deciduous Boreal Tree	1.07	1.17	1.15	1.08	1.13	1.26	1.09	1.12	1.11	1.07	1.12	1.1
BL Evergreen Tropical Tree	1.11	1.17	1.44	1.10	1.15	1.32	1.1	1.17	1.37	1.08	1.13	1.22
BL Evergreen Temperate Tree	1.07	1.23	1.37	1.07	1.23	1.26	1.08	1.21	1.27	1.08	1.22	1.16
BL Deciduous Tropical Tree	1.55	1.7	1.46	1.37	1.63	1.43	1.26	1.54	1.36	1.37	1.51	1.29
BL Deciduous Temperate Tree	1.08	1.14	1.29	1.07	1.11	1.26	1.08	1.1	1.23	1.08	1.1	1.15
BL Deciduous Boreal Tree	1.02	1.05	1.32	1.03	1.04	1.29	1.04	1.04	1.25	1.03	1.06	1.15
BL Evergreen Shrub	1.21	1.91	2.14	1.21	1.83	2.18	1.11	1.3	2.14	1.04	1.19	1.84
BL Deciduous Temperate Shrub	1.25	1.39	1.64	1.16	1.31	1.68	1.11	1.31	1.51	1.07	1.17	1.32
BL Deciduous Boreal Shrub	1.12	1.05	1.14	1.08	1.05	1.14	1.06	1.05	1.12	1.05	1.03	1.1
C3 arctic grass	1.06	1.05	1.19	1.04	1.03	1.21	1.03	1.02	1.16	1.05	1.03	1.17
C3 non-arctic grass	1.38	1.18	1.63	1.30	1.14	1.57	1.19	1.12	1.47	1.23	1.14	1.48
C4 grass	1.01	1.04	0.8	1.00	1.01	0.93	0.97	0.99	0.92	1.01	1.02	0.93
Global Mean	1.10	1.10	1.23	1.07	1.07	1.20	1.06	1.06	1.17	1.08	1.09	1.17

Table S2 Area weighted effect size (treatment / control) of the FACE experiment for each of the natural vegetation plant functional types (PFTs). All values taken from annual means at the end of the experiment (2014). Variables shown here include leaf area index (LAI), gross primary productivity (GPP), net primary productivity (NPP) and total vegetation carbon (VEGC) for each version of CLM (4.0, 4.5, and 5.0).
